# Research advances in chronic thromboembolic pulmonary hypertension: from pathological mechanisms to multidisciplinary management

**DOI:** 10.3389/fcvm.2026.1832792

**Published:** 2026-05-25

**Authors:** Juan-Juan Zhang, Yu-Qian Huang, Xue-Kai Liu, Xiao-Long Sun, Xiang Zhong, Chuan Zhou, Chao Wang, Ping Xie

**Affiliations:** 1 School of Traditional Chinese and Western Medicine, Gansu University of Chinese Medicine, Lanzhou, China; 2 Center of Medical Cosmetology, Chengdu Second People's Hospital, Chengdu, China; 3 The First Clinical Medical College, Gansu University of Chinese Medicine, Lanzhou, China; 4 Department of Geriatric General Surgery, Sichuan Provincial People's Hospital, University of Electronic Science and Technology of China, Chengdu, Sichuan, China; 5 Department of Urology, Shaanxi Provincial People's Hospital, Xi'an, China; 6 Department of Cardiovascular Medicine, Gansu Provincial People's Hospital, Lanzhou, China; 7 The First School of Clinical Medicine, Lanzhou University, Lanzhou, China; 8 Construction Unit of a Branch Center of the National Clinical Research Center for Cardiovascular Diseases, Lanzhou, China

**Keywords:** chronic thromboembolic pulmonary hypertension, diagnostic imaging, multimodal therapy, pathophysiology, precision medicine

## Abstract

Chronic Thromboembolic Pulmonary Hypertension (CTEPH), classified as group 4 pulmonary hypertension (PH), is a progressive disease caused by unresolved pulmonary artery thrombi that undergo organization and fibrosis, leading to increased pulmonary vascular resistance, right heart failure, and death. Over the past decade, the understanding, diagnosis, and management of CTEPH have undergone profound transformation. This review aims to summarize and discuss recent advances in CTEPH, focusing on pathophysiological mechanisms, diagnostic innovations, therapeutic evolution, and future directions. Current evidence establishes CTEPH as a complex, multifactorial disease involving genetic susceptibility, endothelial dysfunction, inflammation, and aberrant vascular remodeling—far beyond simple mechanical obstruction. In diagnosis, novel imaging modalities including ultra-high-resolution CT, dual-energy CT, computational fluid dynamics, and artificial intelligence have significantly enhanced the sensitivity, objectivity, and functional assessment of pulmonary vascular lesions. Therapeutically, a “three-pillar” paradigm is now firmly established, with pulmonary endarterectomy (PEA) as the curative cornerstone, complemented by balloon pulmonary angioplasty (BPA) and targeted pharmacotherapy (e.g., riociguat). This paradigm is increasingly evolving toward multimodal combination strategies, including preoperative bridging therapy and management of residual PH after intervention. Despite these advances, critical challenges remain: precise identification of operable patients, optimization of surgical and interventional techniques, development of novel targeted therapies, and construction of individualized prognostic models integrating multiomics and artificial intelligence. By addressing these core issues, this review provides a comprehensive, clinically oriented perspective on the current state and future trajectory of CTEPH research and multidisciplinary management, while also discussing emerging precision medicine approaches (e.g., multi-omics and artificial intelligence) that remain investigational.

## 1 Introduction

Chronic Thromboembolic Pulmonary Hypertension (CTEPH) is a distinct subtype of pulmonary hypertension characterized by obstruction of the pulmonary arterial system by unresolved organized thrombi and fibrotic material, leading to progressive elevation of pulmonary vascular resistance and ultimately resulting in right heart failure and death (1). Although acute pulmonary embolism (PE) is its primary antecedent event, only a small proportion (approximately 0.4%–3.8%) of patients with acute PE eventually develop CTEPH (2, 3). Nevertheless, given the high incidence of acute PE, the actual disease burden of CTEPH remains substantial.

For many years, CTEPH has been considered a “curable” form of pulmonary hypertension, largely owing to the excellent outcomes associated with pulmonary endarterectomy (PEA). However, with growing insight into the disease, it has become evident that the pathophysiology of CTEPH extends far beyond simple “mechanical obstruction.” In recent years, the refinement of balloon pulmonary angioplasty (BPA) and the approval of targeted medical therapies have markedly expanded the therapeutic landscape for CTEPH and significantly improved patient outcomes (4). Concurrently, the integration of cutting-edge technologies—such as genomics, proteomics, computational fluid dynamics (CFD), and artificial intelligence (AI)—is reshaping our understanding and approach to the diagnosis, classification, and prognostic evaluation of CTEPH from multiple dimensions.

This review aims to summarize and discuss recent advances in the pathological mechanisms, diagnostic assessment, therapeutic strategies, and prognostic management of CTEPH based on recently published high-quality studies. Where appropriate, we distinguish between established clinical tools and emerging experimental approaches (e.g., multi-omics profiling, AI-based predictive models, computational fluid dynamics) that are not yet ready for routine clinical application. Current challenges and future directions in the field are also discussed.

## 2 Pathophysiological mechanisms: from unresolved thrombi to distal microvascular disease

**Figure 1:**
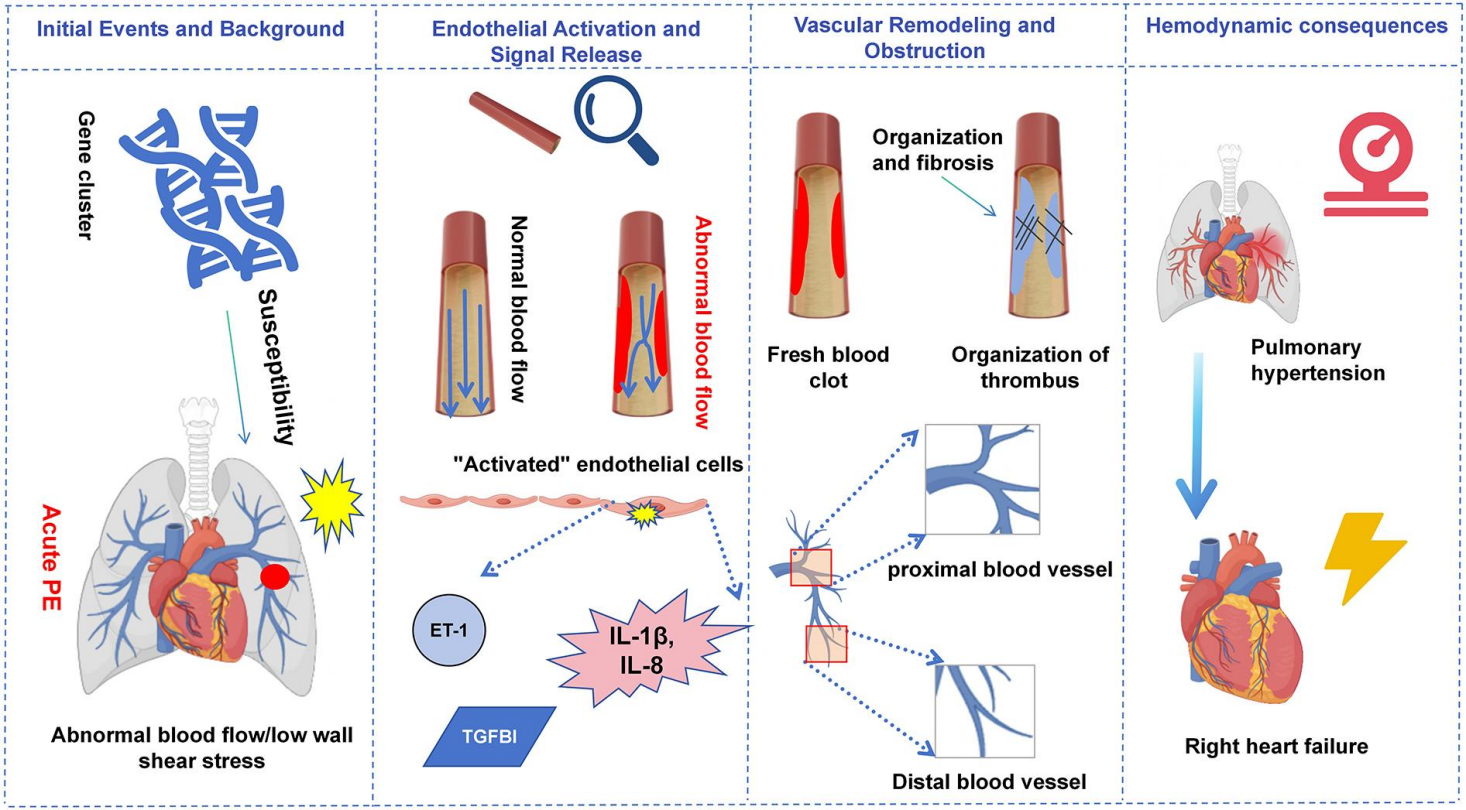
Pathophysiological mechanism network of CTEPH.

Traditionally, CTEPH was considered a consequence of failed thrombus resolution following acute PE. However, the question of why thrombus resolution proceeds smoothly in the vast majority of patients while a minority progress to CTEPH remains unresolved. Recent studies have revealed a more complex mechanistic network (Figure 1, Created by BioRender.com), primarily involving the following aspects:

### 2.1 Genetic susceptibility and thrombotic tendency

Patients with CTEPH exhibit heterogeneity in their genetic background. A single-center genetic analysis identified rare variants in multiple genes related to coagulation, fibrinolysis, platelet function, and vascular disease (e.g., coagulation factor XII (F12), coagulation factor V (F5), von Willebrand factor (VWF), thrombomodulin (THBD), activin A receptor like type 1 (ACVRL1), ring finger protein 213 (RNF213) in CTEPH patients, but no single dominant gene was identified (5). This supports the hypothesis of a “polygenic minor-effect” genetic model, in which subtle defects in multiple genes act in concert with environmental factors (e.g., splenectomy, inflammation) to promote persistent thrombus and abnormal vascular remodeling. A history of splenectomy has been established as a significant risk factor for CTEPH (OR 4.3–5.3) and may influence disease progression by altering the clinical presentation of acute PE (e.g., more distal embolization, less deep vein thrombosis) (6).

### 2.2 Vascular endothelial dysfunction and inflammatory response

Organized thrombi are not inert structures but rather active pathological entities. Studies have demonstrated significantly elevated mRNA and protein expression of endothelin-1 (ET-1) in pulmonary endarterectomy (PEA) specimens from CTEPH patients, which correlated positively with pulmonary vascular resistance (7). This suggests that organized thrombi may serve as an important source of ET-1, contributing to the development and maintenance of PH through its potent vasoconstrictive and pro-proliferative effects. Moreover, endothelial cells derived from PEA specimens (CTEPH-ECs) exhibit a pro-inflammatory phenotype even under basal conditions, with upregulation of interleukin (IL)-8, IL-1β, and MCP-1, associated with sustained activation of the nuclear factor κB (NF-κB) signaling pathway (8). This “inflammatory endothelium” may facilitate fibrotic remodeling of thrombi and promote distal microvasculopathy.

The identification of transforming growth factor-β-induced protein (TGFBI/BIGH3) has provided new insights into thrombus non-resolution. Research has shown that endothelial overexpression of TGFBI delays venous thrombus dissolution in mice, while plasma TGFBI levels are elevated in CTEPH patients and decrease following PEA (9). These findings suggest that TGFBI may impede thrombus organization and recanalization by promoting fibrosis.

### 2.3 Hemodynamic alterations and vascular remodeling

Obstruction of proximal pulmonary arteries leads to severe heterogeneity in pulmonary blood flow distribution and abnormal wall shear stress. Computational fluid dynamics (CFD) studies have demonstrated that time-averaged wall shear stress (TAWSS) in the proximal pulmonary arteries of CTEPH patients is significantly reduced and strongly negatively correlated with the degree of V/Q mismatch (*ρ* = –0.673) (10). Abnormal shear stress itself can induce endothelial dysfunction, characterized by the expression of pro-inflammatory, pro-thrombotic, and pro-angiogenic genes (11). Three-dimensional microfluidic stenosis models constructed from patient CT pulmonary angiography data have confirmed that different degrees of stenosis elicit distinct endothelial gene expression profiles; notably, the transcriptional profile of cells from the 80% stenosis model closely resembled that of CTEPH PEA specimens (11). These findings reveal that hemodynamic changes directly drive vascular biological responses, thereby bridging macroscopic obstruction and microscopic pathology.

### 2.4 Distal microvascular disease

Even after successful relief of proximal obstruction (via PEA or BPA), some patients exhibit persistent PH, attributable to distal microvascular disease (microvasculopathy). This pathology resembles that seen in pulmonary arterial hypertension (PAH) and involves vasoconstriction, intimal hyperplasia, and medial hypertrophy. Studies indicate that circulating levels of angiopoietin-2 (Ang2) are significantly higher in CTEPH patients than in those with chronic thromboembolic disease without PH (CTEPD) and correlate with pulmonary hemodynamic parameters (12). Ang2 is a marker of vascular instability and inflammation; its elevation likely reflects widespread dysfunction of the distal microvascular bed. Thus, CTEPH can be conceptualized as a “dual vascular bed disease”—in which proximal organized thrombi and distal microvasculopathy jointly determine disease severity and prognosis.

## 3 Innovations in diagnostic techniques: from anatomical imaging to functional and intelligent assessment

**Figure 2:**
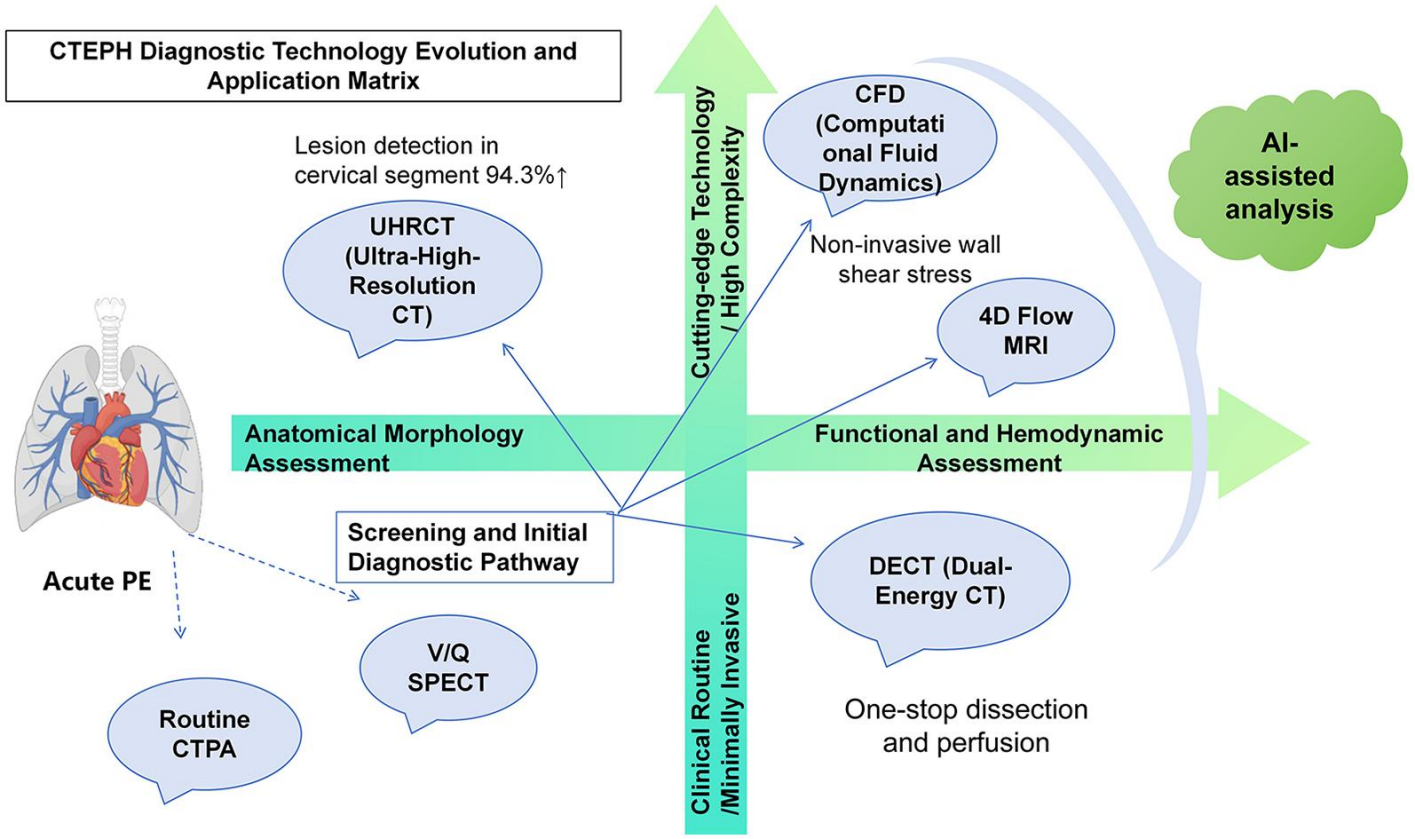
Evolution and application matrix of CTEPH diagnostic techniques.

Early and accurate diagnosis is a prerequisite for the effective management of CTEPH. The current diagnostic workflow relies on imaging evidence of perfusion defects and confirmation of PH by right heart catheterization (RHC) (Figure 2). Recent advances are primarily reflected in the following aspects (Table 1):

**Table 1:** Comparison of advances in diagnostic techniques for CTEPH.

Technology Category	Specific Technique	Core Advantages	Clinical Value & Recent Advances	Limitations/Challenges
Perfusion & Anatomical Imaging	V/Q SPECT/Planar Scintigraphy	Gold standard for screening, sensitivity ∼100%	Defined diagnostic cutoff (≥2.5 segmental mismatched perfusion defects) (13)	Cannot distinguish acute from chronic thrombi; radiation exposure; limited sensitivity for subsegmental lesions
	Conventional CTPA	Assesses thrombus anatomy, cardiac morphology	Basic diagnostic tool	Insufficient sensitivity for distal thrombi
	Ultra-High-Resolution CT (UHRCT)	Sub-millimeter spatial resolution	Markedly improves detection of subsegmental lesions (94.3% vs 54.6%); precise BPA planning (14)	Slightly higher radiation dose; not yet widely available
	Dual-Energy CT (DECT) + Iodine Map	One-stop assessment (anatomy + perfusion)	Excellent diagnostic performance (sensitivity 100%, specificity 96.6%); potential to replace V/Q scintigraphy (15)	Requires specific equipment; iodine map interpretation requires experience
Functional & Hemodynamic Assessment	Computational Fluid Dynamics (CFD)	Noninvasive quantification of hemodynamic parameters (e.g., wall shear stress)	TAWSS is a sensitive biomarker for chronic thrombotic burden and perfusion mismatch (10)	Model construction complex; not yet routine in clinical practice
	4D Flow MRI	Noninvasive assessment of pulmonary artery flow patterns	Vortex duration strongly correlates with mPAP; potential for noninvasive PH estimation (16)	Long acquisition time; high cost; sensitive to motion artifacts
	Hyperpolarized ^12^⁹Xe MRI	Assesses microvascular gas exchange in lung parenchyma	Quantifies cardiopulmonary oscillatory defects; reflects microvascular function; monitors treatment response (17)	Highly research-oriented; extremely limited accessibility
Artificial Intelligence & Automation	AI-Assisted Image Analysis	Automated, quantitative, objective	Automatic quantification of pulmonary perfusion abnormalities; correlates with hemodynamic parameters (19); CTEPH screening using only NCCT (20)	Model generalizability needs validation; clinical integration still in progress
	Machine Learning Predictive Models	Integrates multi-dimensional data to predict outcomes	Predicts BPA efficacy (AUC 0.865) (21); CT-based feature risk stratification (AUC 0.82) (22)	Requires large, high-quality datasets for training

### 3.1 Advances and comparisons in perfusion imaging

Ventilation/perfusion (V/Q) scintigraphy, particularly single-photon emission computed tomography (SPECT), remains the gold standard for CTEPH screening, with a sensitivity approaching 100% (13). Studies have further defined the diagnostic cutoff for planar V/Q scanning: ≥2.5 segmental mismatched perfusion defects yield optimal diagnostic performance (sensitivity 100%, specificity 94.7%) (13). However, V/Q scintigraphy cannot distinguish between acute and chronic thrombi and has limited sensitivity for subsegmental lesions.

Computed tomography pulmonary angiography (CTPA) is indispensable for assessing vascular anatomy, thrombus location, and cardiac morphology. Ultra-high-resolution CT (UHRCT, detector width 0.25 mm) substantially improves the detection sensitivity for subsegmental branch lesions from 54.6% with conventional CT to 94.3%, and enables more accurate classification of lesion morphology (e.g., webs, nets), greatly facilitating preoperative planning for BPA (14). Dual-energy CT (DECT), combined with iodine mapping, simultaneously provides information on vascular anatomy and lung parenchymal perfusion. Studies have shown that DECT achieves a sensitivity of 100% and a specificity of 96.6% for diagnosing CTEPH, with high concordance (92.1%) with V/Q-SPECT in segmental classification (15). This positions DECT as a potential one-stop diagnostic tool.

### 3.2 Functional imaging and noninvasive hemodynamic assessment

Conventional imaging primarily evaluates morphological changes, whereas emerging technologies are dedicated to the noninvasive assessment of pulmonary vascular function and hemodynamics.

1. Computational Fluid Dynamics (CFD): CFD models based on CTPA enable calculation of parameters such as pulmonary artery wall shear stress. Studies have confirmed that proximal pulmonary artery TAWSS is a sensitive indicator of chronic thromboembolic burden, effectively differentiating CTEPH, CTEPD, and control groups, and is strongly correlated with the degree of V/Q mismatch, offering the potential for early identification of subclinical perfusion abnormalities (10).
2. Cardiac Magnetic Resonance (CMR) and 4D Flow: CMR is the gold standard for assessing right ventricular function and myocardial characteristics. 4D flow imaging quantifies vortical flow within the pulmonary artery. Research has shown that the duration of vortical flow in the main pulmonary artery is highly correlated with mPAP (r = 0.805), and using a cutoff of 8.6% enables detection of PH with high sensitivity (16). Furthermore, hyperpolarized 129Xe gas-exchange MRI allows quantification of cardiopulmonary oscillatory signals, which are impaired in CTEPH patients and improve after pulmonary endarterectomy, potentially reflecting impaired microvascular flow (17).
3. Positron Emission Tomography (PET): 18F-FDG PET has demonstrated significantly higher FDG uptake in the right ventricle and right atrium of CTEPH patients compared to controls, correlating with mPAP and right atrial pressure, suggesting that myocardial metabolic abnormalities are associated with disease severity (18).

### 3.3 Artificial intelligence and automated quantitative analysis

AI is transforming the paradigm of image analysis and disease prediction.

1. Automated Quantification: Machine learning models based on Bayesian analysis can automatically quantify perfusion abnormalities on CTPA, distinguishing the proportions of lung parenchyma with high, normal, and low perfusion. These parameters significantly correlate with mPAP, pulmonary vascular resistance, BNP, and mosaic attenuation, thereby providing objective and reproducible assessment metrics (19)
2. Diagnostic Assistance: An AI model employing a cascade network and multiple-instance learning framework enables automated diagnosis of CTEPH using only non-contrast CT (NCCT), without the need for precise lesion annotation, offering a novel approach for early screening (20). It should be noted, however, that these AI models remain at the proof-of-concept or early validation stage; prospective studies and regulatory approvals are required before they can be integrated into routine clinical practice. Critically, while these AI-driven approaches offer objectivity, their current evidence base is limited by:(1) almost exclusive reliance on single-center, retrospective datasets with inherent selection bias; (2) lack of external validation across different CT protocols and patient demographics; (3) ‘black-box’ nature limiting clinical interpretability; and (4) absence of prospective trials demonstrating incremental benefit over expert human reading. Therefore, at present, AI should be viewed as a supplementary decision-support tool rather than a standalone diagnostic modality for CTEPH.
3. Therapeutic Outcome Prediction: Machine learning models integrating clinical and echocardiographic parameters (e.g., proportion of occlusive lesions, TAPSE/PASP ratio, 6MWD) effectively predict patient response to BPA, with the logistic regression model demonstrating the best predictive performance (AUC 0.865) (21). Another study successfully predicted hemodynamic risk stratification using CT features (e.g., contrast medium reflux into the hepatic veins, mosaic attenuation, right atrial/left ventricular area ratio) via a random forest model (AUC 0.82) (22).

### 3.4 Biomarkers: from single indicators to combined models

Conventional biomarkers such as BNP/NT-proBNP have well-established roles in prognostic assessment. Recent studies have explored the value of combined indicators and novel biomarkers.

1. Combined Indicators: The NT-proBNP/albumin ratio (NTAR) outperforms NT-proBNP alone in predicting hospitalization duration and mortality in patients with PAH and CTEPH (23)
2. Novel Biomarkers: Soluble ST2 is elevated in patients with acute PE and shows predictive value for the subsequent development of CTEPH (AUC 0.783) (24). Levels of mid-regional pro-atrial natriuretic peptide (MR-proANP) and copeptin correlate with CTEPH disease severity and decrease after effective treatment, making them useful for assessing therapeutic response (25). Moreover, exercise-induced MR-proANP can unmask latent right heart failure in patients with normal resting right atrial pressure (26).

## 4 Diversification and individualization of treatment strategies: deepening the “three modalities” framework

**Figure 3:**
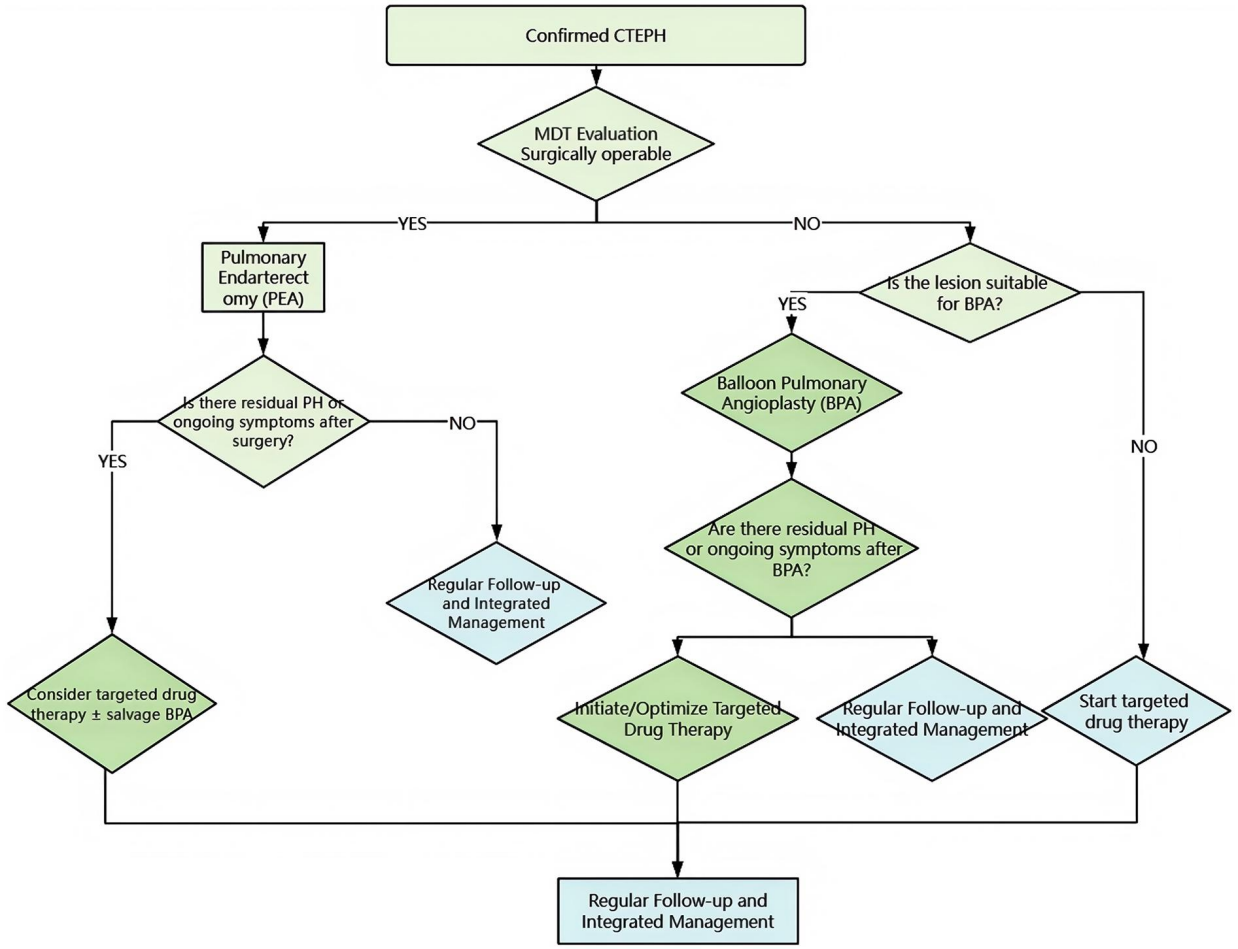
Individualized treatment decision pathway for CTEPH.

The therapeutic goals for CTEPH are to maximize relief of vascular obstruction, reduce pulmonary vascular resistance, and improve right heart function and clinical symptoms. Pulmonary endarterectomy (PEA), balloon pulmonary angioplasty (BPA), and targeted medical therapy constitute the three mainstays of contemporary treatment. The individualized decision-making pathway is summarized in Figure 3.

### 4.1 Surgical pulmonary endarterectomy (PEA): refinement and expansion of the gold standard

PEA remains the first-line curative treatment for operable patients. The second international CTEPH registry reported a 3-year survival rate of 94% in patients undergoing PEA, an improvement from 89% in the 2007–2012 registry data (4).

1. Broadening of Surgical Indications: Age and comorbidities are no longer absolute contraindications. Studies have shown that even elderly patients (≥70 years or ≥80 years) who undergo PEA at experienced centers achieve significant hemodynamic and functional improvements, with perioperative mortality comparable to that of younger patients (∼2%) (27, 28). For patients with unilateral CTEPH/CTEPD, PEA is also safe and effective, markedly improving symptoms and hemodynamics (29).
2. Optimization of Perioperative Management: Intraoperative transesophageal echocardiography (TEE) plays a key role in identifying occult thrombi (e.g., in the inferior vena cava) and guiding surgical decision-making (30). A novel strategy of adding 5% human albumin to the cardiopulmonary bypass (CPB) prime solution significantly reduces intraoperative positive fluid balance, decreases vasoactive-inotropic scores, and shortens operative time and hospital stay (31).
3. Assessment of Postoperative Residual PH: Residual PH after surgery is an important determinant of long-term prognosis. Although electrocardiogram-derived ventricular gradient for right ventricular pressure overload (VG-RVPO) changes correlate with alterations in mPAP and right ventricular mass, it is not yet sufficiently reliable to rule out residual PH; RHC remains the gold standard for evaluation (32).

### 4.2 Balloon pulmonary angioplasty (BPA): a mainstay of interventional therapy

BPA has become the mainstream option for patients who are ineligible for PEA (due to unfavorable anatomy or comorbidities) or who have residual PH after surgery.

1. Efficacy and Safety: BPA significantly improves hemodynamics (reducing mPAP and PVR), exercise capacity, WHO functional class, and quality of life, with durable effects (27, 33). A *post-hoc* analysis of the RACE trial revealed that BPA primarily reduces right ventricular afterload by lowering mPAP, thereby improving right ventricular function; in contrast, riociguat lowers PVR mainly by increasing cardiac output, with only limited effects on right ventricular function (34).
2. Technical Advances: Imaging modalities such as UHRCT and cone-beam CT have substantially enhanced preoperative planning and intraoperative guidance for BPA, facilitating the identification of more distal and complex lesions (14, 35).
3. Special Populations: Elderly patients: BPA achieves hemodynamic improvements in elderly patients (≥70 years) comparable to those of PEA, with potential advantages in improving WHO functional class (27). Importantly, accumulating evidence demonstrates that BPA is equally safe and effective in older patients as in their younger counterparts. Velázquez Martín et al. reported that in CTEPH patients aged ≥70 years, BPA significantly reduced mean pulmonary arterial pressure and pulmonary vascular resistance, improved functional class, and reduced the need for PH-targeting medications, with a low rate of procedure-related complications (pulmonary injury in 3.4% of procedures, no grade 5 injuries) and good survival (90.5% at 1 year, 82.8% at 3 years) (36). Similarly, Shinya et al. compared older (≥75 years) and younger (<75 years) CTEPH patients undergoing BPA and found comparable improvements in hemodynamics, 6-minute walking distance, and pulmonary diffusion capacity in both groups, with no in-hospital mortality or need for extracorporeal membrane oxygenation in either group [Ref2]. Notably, while all-cause mortality during follow-up was higher in the older group, none of the deaths were related to pulmonary hypertension, suggesting that BPA itself does not increase PH-specific mortality risk in elderly patients (37).

Patients with elevated pulmonary artery wedge pressure (PAWP): Elevated PAWP, often indicative of concomitant left ventricular dysfunction, represents an emerging special population. A recent retrospective analysis by Szwed et al. of 170 CTEPH patients undergoing BPA found that elevated PAWP (present in 13.5% of the cohort) was associated with an older age, higher BMI, elevated baseline troponin levels, and a greater burden of comorbidities (diabetes, atrial fibrillation, deep vein thrombosis, chronic kidney disease) [Ref3]. Following BPA, the elevated PAWP group showed significant, yet significantly smaller, improvements in mean pulmonary arterial pressure (*p* = 0.026), pulmonary arterial compliance (*p* = 0.002), and 6-minute walking distance (*p* = 0.011) compared to the normal PAWP group (38). Importantly, there were no significant differences between the two groups in overall survival or adverse event rates [Ref3]. These findings suggest that while the clinical benefits of BPA may be attenuated in patients with elevated PAWP, the procedure remains a feasible and safe option in this subgroup, with careful periprocedural management.

### 4.3 Targeted pharmacological therapy: evolving role and updated evidence

Pharmacological therapy is primarily indicated for patients who are inoperable or ineligible for intervention, or who have residual PH after surgery.

1. Riociguat: As a soluble guanylate cyclase (sGC) stimulator, riociguat is currently the only targeted drug approved for the treatment of CTEPH. Multiple real-world studies have confirmed that riociguat effectively improves functional class, 6-minute walking distance, and BNP levels in CTEPH patients (39). Even in very elderly patients (≥80 years) or those with risk factors for heart failure with preserved ejection fraction (HFpEF), riociguat demonstrates favorable tolerability and efficacy (40). A long-term observational study showed that riociguat provides sustained improvements in PVR and cardiac index for up to 8 years (41). Furthermore, in patients who remain symptomatic after completing BPA, switching from a phosphodiesterase-5 inhibitor (PDE5i) to riociguat further improves pulmonary hemodynamics and functional status, although attention to the risk of adverse events is warranted (42).
2. Prostacyclin Pathway Agents: These include prostacyclin analogues and the oral prostacyclin receptor agonist (e.g., selexipag). In a randomized controlled trial (SELECT trial) involving patients with operable or inoperable CTEPH who had persistent/recurrent PH after surgery, selexipag failed to significantly improve the primary endpoint of PVR, and the study was terminated early due to futility (43). However, a meta-analysis of six RCTs demonstrated that prostacyclin pathway agents significantly improve PVR, right atrial pressure, cardiac index, and WHO functional class, suggesting that they may still hold value in certain subgroups (e.g., those with predominantly distal microvascular disease) (44).
3. Anticoagulation: Lifelong anticoagulation is the cornerstone of management for all CTEPH patients, aimed at preventing recurrence. Both vitamin K antagonists (VKAs) and direct oral anticoagulants (DOACs) are used; the second international CTEPH registry reported no difference in survival between the two (4). The choice of anticoagulant should consider drug-drug interactions (e.g., with riociguat), comorbidities, and patient preference (45).

### 4.4 Multimodal treatment and individualized decision-making

**Figure 4:**
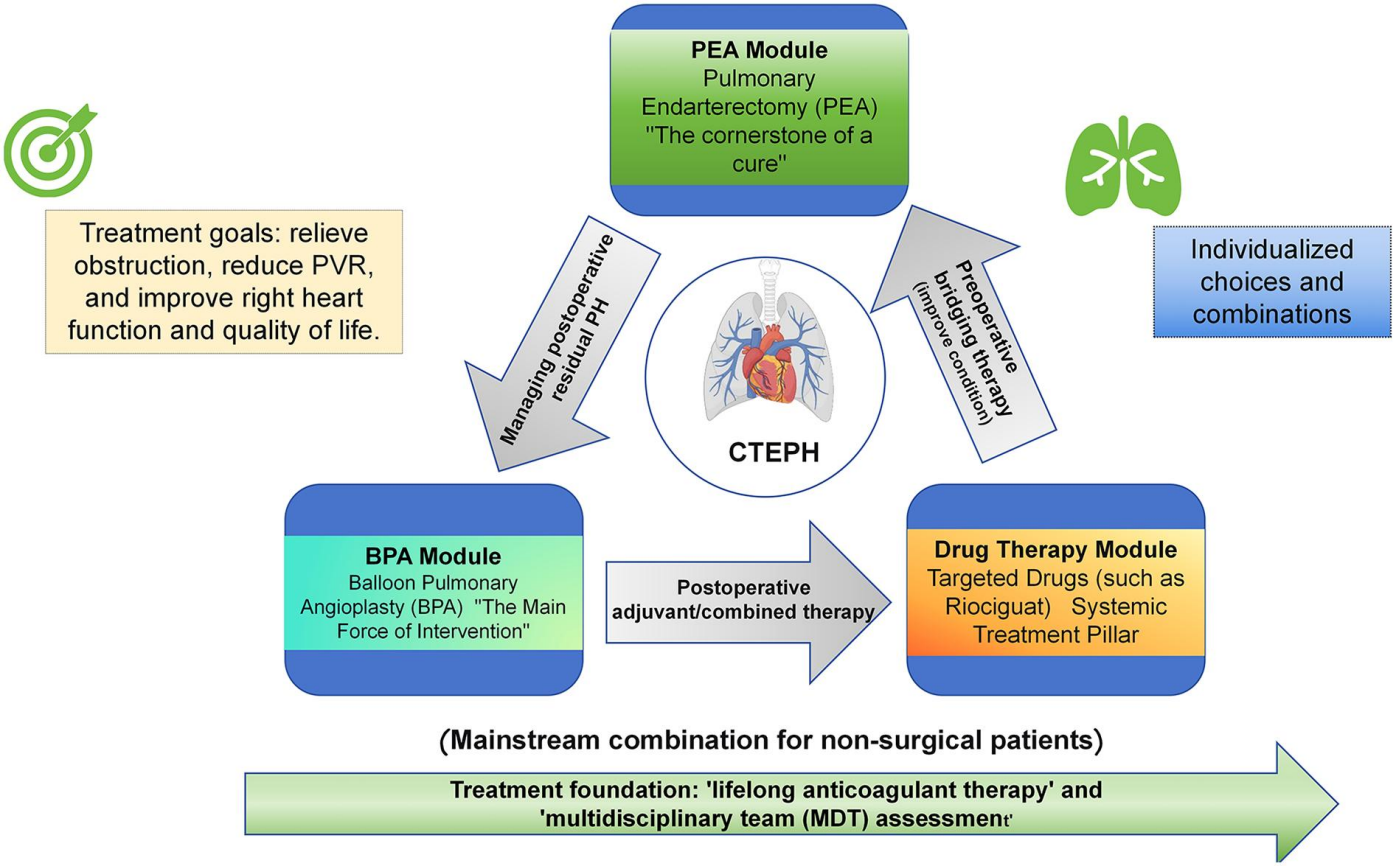
Schematic diagram of multimodal combination therapy for CTEPH.

In clinical practice, PEA, BPA, and pharmacotherapy are often applied in combination as a “multi-modality” approach (Figure 4). Examples include preoperative use of medication to optimize patient status (bridging therapy) or adding pharmacotherapy for residual PH after PEA or BPA. The CLARITY global survey revealed substantial heterogeneity in treatment selection across centers and regions, reflecting the current gap between guidelines and practice, as well as individualized decision-making based on center experience and resource availability (46).This heterogeneity reflecting not only the gap between guidelines and practice but also a fundamental lack of high-level evidence to resolve several clinical dilemmas:（1）What is the optimal sequence for patients with operable CTEPH but significant distal disease? Preoperative pharmacotherapy (‘bridging’) remains empirically driven.（2）How to define ‘operability’ objectively? Current criteria vary markedly between expert centers, introducing substantial selection bias into outcome comparisons.（3）For residual PH after PEA or BPA, should treatment escalation follow PAH algorithms? Direct evidence for this common practice is surprisingly lacking.

These unresolved questions underscore that the ‘three-pillar’ paradigm, while established, operates within a large zone of clinical uncertainty, necessitating individualized decision-making and further randomized trials.

## 5 Prognostic assessment, quality of life, and economic burden

With significant advances in treatment, the survival of patients with CTEPH has greatly improved, and the focus has shifted toward functional status, quality of life, and long-term prognosis.

### 5.1 Prognostic assessment tools

In addition to traditional hemodynamic parameters, comprehensive risk assessment models such as COMPERA 2.0 have been widely adopted. Studies have found that patients who fail to achieve low-risk status after one year of treatment tend to be older and have more comorbidities (47). Right ventricular strain analysis, derived from cardiac magnetic resonance feature tracking, is an emerging marker for assessing right ventricular mechanical function; it improves significantly after BPA and correlates well with hemodynamic and myocardial remodeling parameters (48).

### 5.2 Quality of life

Studies have demonstrated that both PEA and BPA lead to significant and comparable improvements in quality of life at six months post-procedure (49). Dyspnea (NYHA class) and post-exertion fatigue (Borg score) are the clinical parameters most strongly associated with quality of life (46). The disease-specific quality-of-life instrument PAH-SYMPACT™ has also been validated in CTEPH patients and effectively captures disease symptoms and impact (50).

### 5.3 Economic burden

bstantial economic burden on society and patients. A Finnish study reported that targeted PH medications constitute the largest cost component in both PAH and CTEPH (67% and 60%, respectively), followed by hospitalization costs and disability pensions (51). Total costs were significantly lower among CTEPH patients who underwent PEA compared to those who did not, highlighting the economic value of curative interventions (51). Early health economic data on the cost of BPA in France have also been reported (52).

## 6 Current challenges and future directions

Despite considerable progress, numerous challenges remain in the field of CTEPH. Key research hotspots for the future are summarized in Table 2.

**Table 2:** Current challenges and future directions in CTEPH research.

Domain	Current Major Challenges	Future Research Directions & Hotspots
Pathological Mechanisms	1. Decisive molecular mechanisms driving the transition from acute PE to CTEPH remain unclear. 2. Causal relationship and interaction between distal microvasculopathy and proximal thrombi are poorly understood. 3. Lack of specific disease-driving therapeutic targets.	1. Multi-omics integration: Employ genomics, transcriptomics, proteomics, and metabolomics to identify key signaling pathways and biomarkers of disease transition. 2. Advanced disease models: Develop organoids and 3D-bioprinted vascular models to study cell-cell interactions. 3. Focus on novel pathways: Investigate the roles of TGFBI, Ang2, inflammasomes, etc., in fibrosis and vascular remodeling.
Early Diagnosis	1. Lack of efficient, low-cost screening strategies after acute PE. 2. Current imaging techniques insufficient for detecting early/subclinical CTEPD. 3. High subjectivity in assessing “operability”.	1. Develop risk prediction models: Combine clinical factors, biomarkers (e.g., sST2, MR-proANP), and simplified imaging to create screening tools. 2. Promote functional imaging: Validate and disseminate one-stop assessment protocols (e.g., DECT, CFD). 3. AI-empowered decision-making: Use deep learning for objective prediction of operability, BPA efficacy, and prognosis.
Treatment Strategies	1. Some patients respond poorly to current therapies (PEA/BPA/medications). 2. Bottlenecks in targeted drug development (e.g., negative SELECT trial). 3. Lack of high-level evidence on optimal timing and combination of multimodal treatments.	1. Precise patient stratification: Stratify by molecular phenotype and imaging features; conduct umbrella/basket trials. 2. Novel drug development: Target thrombofibrosis (anti-fibrotics), inflammation (specific anti-inflammatory agents), and vascular remodeling. 3. Optimize combination strategies: RCTs on bridging therapy before PEA/BPA and intensified postoperative pharmacotherapy.
Long-term Management	1. Management strategies for elderly patients with multiple comorbidities. 2. Prevention and treatment of long-term complications (e.g., hemoptysis, right heart failure). 3. Insufficient real-world long-term efficacy and health economics data.	1. Establish consensus on geriatric CTEPH management. 2. Long-term registry studies: Collect real-world data to evaluate long-term outcomes and cost-effectiveness of new technologies and treatments. 3. Develop telemonitoring and rehabilitation: Use wearable devices and mobile health to improve self-management and quality of life.

## 7 Conclusion

Research into CTEPH has entered an unprecedented phase of rapid evolution. Our understanding of the disease is shifting from the macroscopic to the microscopic, and from a reductionist view toward a systems-based perspective. Diagnostic technologies are advancing toward greater precision, functional integration, and intelligent automation. The therapeutic landscape has evolved from a PEA-centric model into a multidimensional paradigm in which PEA, BPA, and targeted medical therapy stand as three complementary pillars. In conclusion, CTEPH research has made undoubted strides, transforming a once ‘mechanically obstructive’ disease into a paradigm of multimodal, precision-oriented management. However, a sober appraisal reveals that many promising advances—from CFD biomarkers to AI-based outcome prediction—remain at an early translational stage, lacking the robust prospective validation required for clinical adoption. The failure of the SELECT trial also serves as a cautionary reminder that PAH-pathway drugs may not directly translate to CTEPH. Looking ahead, the field must prioritize not only technological innovation but also rigorous, multicenter, comparative-effectiveness research to resolve current uncertainties regarding patient selection, treatment sequencing, and the true added value of emerging tools. Only then can the goal of truly individualized, high-quality long-term care be realized for all CTEPH patients.

## Glossary

6MWD: 6-minute walking distance
AI: artificial intelligence
Ang2: angiopoietin-2
AUC: area under the curve
BNP: brain natriuretic peptide
BPA: balloon pulmonary angioplasty
CFD: computational fluid dynamics
CMR: cardiac magnetic resonance
CPB: cardiopulmonary bypass
CT: computed tomography
CTEPD: chronic thromboembolic disease
CTEPH: chronic thromboembolic pulmonary hypertension
CTPA: computed tomography pulmonary angiography
DECT: dual-energy computed tomography
DOAC: direct oral anticoagulant
ET-1: endothelin-1
FDG: fluorodeoxyglucose
HFpEF: heart failure with preserved ejection fraction
IL: interleukin
MCP-1: monocyte chemoattractant protein-1
mPAP: mean pulmonary arterial pressure
MR-proANP: mid-regional pro-atrial natriuretic peptide
MRI: magnetic resonance imaging
NCCT: non-contrast computed tomography
NF-*κ*B: nuclear factor kappa-B
NT-proBNP: N-terminal pro-brain natriuretic peptide
NTAR: NT-proBNP/Albumin ratio
NYHA: New York heart association
PAH: pulmonary arterial hypertension
PASP: pulmonary artery systolic pressure
PE: pulmonary embolism
PEA: pulmonary endarterectomy
PDE5i: Phosphodiesterase-5 inhibitor
PET: positron emission tomography
PH: pulmonary hypertension
PVR: pulmonary vascular resistance
RCT: randomized controlled trial
RHC: right heart catheterization
sGC: soluble guanylate cyclase
SPECT: single-photon emission computed tomography
sST2: soluble suppression of tumorigenicity 2
TAPSE: tricuspid annular plane systolic excursion
TAWSS: time-averaged wall shear stress
TEE: transesophageal echocardiography
TGFBI: transforming growth factor-beta-induced protein
UHRCT: ultra-high-resolution computed tomography
V/Q: ventilation/perfusion
VKA: vitamin K antagonist
WHO: World Health Organization
WHO-FC: World Health Organization functional class.
